# CGRP Is Critical for Hot Flushes in Ovariectomized Mice

**DOI:** 10.3389/fphar.2018.01452

**Published:** 2019-01-04

**Authors:** Daniel B. Wilhelms, Hua Dock, Haissa O. Brito, Emma Pettersson, Andrea Stojakovic, Joanna Zajdel, David Engblom, Elvar Theodorsson, Mats L. Hammar, Anna-Clara E. Spetz Holm

**Affiliations:** ^1^Division of Drug Research, Department of Medical and Health Sciences, Linköping University, Linköping, Sweden; ^2^Department of Emergency Medicine, Local Health Care Services in Central Östergötland, Linköping, Sweden; ^3^Department of Clinical and Experimental Medicine, Linköping University, Linköping, Sweden; ^4^Division of Childrens and Womens Health, Department of Clinical and Experimental Medicine, Linköping University, Linköping, Sweden; ^5^Department of Gynaecology and Obstetrics in Linköping, Center of Paediatrics and Gynaecology and Obstetrics, Linköping, Sweden

**Keywords:** calcitonin gene-related peptide, antagonist, thermoregulation, hot flushes, mice

## Abstract

Hot flushes are common and troublesome symptoms of menopause. The neuropeptide calcitonin gene-related peptide (CGRP) is increased in plasma during hot flushes but it has not been clear if CGRP is causally involved in the mechanism underpinning the flushes. Here, we examined the effect of interventions with CGRP in a mouse model of hot flushes based on flush-like temperature increases triggered by forced physical activity in ovariectomized mice. Compared to normal mice, ovariectomized mice reacted with an exaggerated, flush-like, temperature increase after physical exercise. This increase was completely blocked by the non-peptide CGRP-antagonist MK-8825 (-0.41 degrees Celsius, 95% CI: -0,83 to 0,012, *p* < 0.0001) at a dose that had no obvious effects on locomotor activity (50 mg/kg). Further, the flush-like temperature increases were strongly attenuated in ovariectomized mice lacking αCGRP due to a genetic modification. Collectively, our findings suggest that CGRP is an important mediator of experimentally induced hot flushes and they identify CGRP antagonists as promising treatment candidates for women and possibly also men with hot flushes.

## Introduction

Hot flushes are classic menopausal symptoms in women (Hammar et al., 1984; McKinlay et al., 1992) but are also reported by men after castration therapy or with testicular insufficiency (Feldman et al., 1976; Karling et al., 1994), usually persisting over many years and impairing quality of life (Karling et al., 1994). Most subjectively reported hot flushes are also objectively confirmed by increased blood-flow, skin-conductance and skin-temperature (Freedman, 1989; Spetz et al., 2001). The gold standard for treating menopausal symptoms is hormone therapy in the form of estrogen with or without progestogen (Shen and Stearns, 2009), which reduces the flushes by 75% (MacLennan et al., 2004). However, reports during the early 2000s on increased risk of breast cancer and cardiovascular disease (Herrington et al., 2002; Rossouw et al., 2002; Beral, 2003) led to a substantial reduction in hormone therapy use. Despite the fact that later studies suggest that the risks are smaller in recently menopausal women than previously believed (Phillips and Langer, 2005; Rossouw et al., 2007) alternative treatments for menopausal symptoms are needed, especially for women with a history of breast cancer or thromboembolic disease. There are a few alternative pharmacological and non-pharmacological treatments, but none of these seem to be as effective as hormone therapy (Wyon et al., 2004; Albertazzi, 2007; Wong et al., 2009; Daley et al., 2011). Hot flushes usually correlate with the acute decrease in estrogen levels during the menopausal transition. Although the physiological mechanisms underpinning hot flushes are not fully elucidated, changes in the thermoregulatory center in the hypothalamus that make the thermoneutral zone very narrow is a key component (Sturdee et al., 2017). Specifically, a recent study has identified kisspeptin and neurokinin B neurons in the arcuate nucleus of the hypothalamus as key players in the occurrence of thermoregulatory instability (Padilla et al., 2018). As a result, even very small increases in core body temperature trigger heat dissipation responses in order to decrease central temperature (Freedman and Subramanian, 2005; Stearns, 2007). This response includes skin vasodilation and sweat gland activation, and it precedes the subjective sensation of heat suggesting that it underpins the subjective sensations of the flush rather than being a consequence of the perceived heat. However, our understanding of the molecular mechanisms behind hot flushes is highly limited and this has hampered the development of rational treatment alternatives.

The neuropeptide calcitonin gene-related peptide (CGRP), which is found predominantly in sensory C and Aδ nerve fibers, has been suggested to be involved in hot flushes (Wyon et al., 2000; Spetz et al., 2001; Oliveira et al., 2018). CGRP is a potent dilator of the microvasculature and enhances cholinergic sweating in rats and possibly also in humans. CGRP has been detected in human sudomotor cholinergic nerves stimulating eccrine sweat glands (Kennedy et al., 1994) and likely influences sweating under physiological conditions. When CGRP was administered intravenously to healthy male volunteers it caused symptoms similar to hot flushes, with a dose-dependent increase in cutaneous blood flow (Jernbeck et al., 1990). CGRP has been shown to increase in plasma during hot flushes in postmenopausal women and men castrated due to prostatic carcinoma (Chen et al., 1993; Valentini et al., 1996; Wyon et al., 2000; Spetz et al., 2001).

Despite the correlational data linking CGRP and hot flushes, no attempts have been made to determine if blocking the actions of CGRP inhibits flushes. The pharmacological tools are available; antagonists toward CGRP-receptors have been developed and tested in human phase II and phase III studies, mainly for treatment of migraine (Villalon and Olesen, 2009; Bell et al., 2010; Hirsch et al., 2013) and MK-8825, a non-peptide CGRP receptor antagonist administered orally, has been shown to be active in rodents migraine (Villalon and Olesen, 2009). In order to find out if CGRP plays a causal role in hot flushes and if CGRP antagonism has therapeutic potential for this indication, we used the CGRP receptor antagonist MK-8825 in a previously reported mouse model of menopausal hot flushes (Shuto et al., 2005).

## Materials and Methods

### Animals

All animal procedures were performed according to the practices of the Swedish Board of Animal Research and were approved by the Regional Ethical Committee for Animal Care and Use, Linkoping, Sweden. All possible efforts were made to minimize suffering. Animals were housed at Linköping University Animal Department with free access to water and standard rodent chow at a room temperature of 24 ± 1°C with 12-h light/dark cycles. For pharmacological experiments, Imprinting Control Region (ICR, CD-1) female mice weighing 25–30 g were used. The mice lacking αCGRP and their littermate controls were from MMRRC (RRID:MMRRC_036773-UNC) and were on a C57Bl/6 background and weighed 18–26g (McCoy et al., 2013). All mice were between 12 and 20 weeks of age at the start of the experiments.

### Experimental Protocol

**FIGURE 1:**
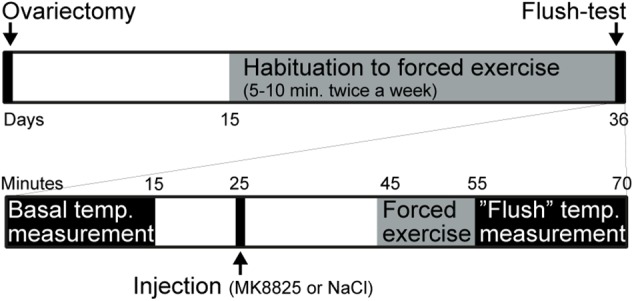
Summary of the experimental design. The upper part summarizes the entire experiment and the lower part shows the test-day in more detail.

Mice were randomly assigned to a sham group or an ovariectomized group. The mice in the ovariectomized group underwent bilateral ovariectomy under isoflurane anesthesia at 4.5% for induction and 1.5% for maintenance in a mixture (30/70%) of oxygen/nitrous oxide at day 1. Beginning on day 15, a program was started in which mice were trained to run on a motor-driven treadmill (AccuPacer, Accuscan Instruments, United States) for 3 weeks. During the first week mice were allowed to run at 4 m/min for 1 min and then 7 m/min for 5 min each once a week. During the second week, mice were allowed to run at 7 m/min for 5 min and then 15 m/min for 5 min each once a week. During the third week mice ran at 15 m/min for 10 min twice a week. Thirty-five days after the ovariectomy, mice were subjected to treatments and measurement. Mice that ran less than 10 min or were not willing to go inside the mouse-holder were excluded from the experiment. An overview of the experimental design is presented in Figure 1.

### Drug Administration

The ovariectomized mice were randomly allocated to treatment with vehicle (saline) or the CGRP-antagonist MK-8825 (Merck, United Kingdom). Twenty minutes before running, 200 μl of sodium chloride (0.9%; B. Braun, Germany) or CGRP-antagonist MK-8825 at a dose of either 10 mg/kg body weight or 50 mg/kg body weight of (8750 μg/ml in 0.9% NaCl) was injected intraperitoneally.

### Measurement and Data Analysis of Tail-Skin Temperature

Tail-skin temperature was measured as previously described [28]. In brief, mice were restrained in a holder and tail-skin temperature was measured at the dorsal surface of the tail one cm from its base with a thermotracer (Infrared TH2640, NEC Avio, Tokyo, Japan) for 15 min, 30 min before exercise on the treadmill. The mean tail-skin temperature during the first 6-min period was defined as basal tail-skin temperature. The mice were then injected with either saline or MK-8825 (50 mg/kg of body weight) and, 20 min after the intraperitoneal injection, allowed to run for 10 min (15 m/min) on a motor-driven treadmill (AccuPacer, Accuscan Instruments, United States). Tail-skin temperature was measured for 15 min after the exercise, and changes in tail-skin temperature at 1–6 min after exercise were assessed. The change in tail-skin temperature was calculated by subtracting the basal tail-skin temperature (see above) from the mean tail-skin temperature after exercise. All measurements of tail-skin temperature were performed between 9:00 and 12:00 h and the room temperature was maintained at 22–25°C throughout the measurement period.

### Open Field Test

Mice were placed in an open-field arena (45 cm x 45 cm), and their activity was recorded for 15 min with a video camera placed above the arena. EthoVision tracking software (Noldus Information Technology bv, Wageningen, Netherlands) was used for data processing and analysis. Total distance traveled, and the time mice spent in the center of the arena was quantified.

### Immunohistochemistry

Mice were perfused with saline followed by 4% paraformaldehyde in PBS. The tissue was postfixed in the same solution for 4 h in 4°C and placed in 30% sucrose till saturated. 40 μm free floating sections were blocked in 10% Normal Donkey Serum in PBST (PBS and 0,4% Triton-X) for 24 h. After blocking, sections were incubated in a mixture of primary antibodies (rabbit anti-CGRP, 1: 5 000, PenLab, T-4031 and chicken anti-GFP, 1:10 000, Abcam, ab13970) over night. The next day sections were rinsed 5 times with PBST and moved to a mixture of secondary antibodies: goat anti-chicken Alexa Fluor 488, 1:1000 and donkey anti-rabbit Alexa Fluor 568, 1:1000. After 2 h the sections were rinsed in PBST and mounted in ProLong^®^Gold Antifade (ThermoFisher). The images were obtained on fluorescent microscope (Nikon Eclipse E800).

### Statistical Analysis

All data were represented as the mean ± SEM. Students *T*-test was used when comparing two groups and 1-way ANOVA followed by Holm-Sidaks multiple comparisons test was used when comparing more than two groups. Differences at the *p* < 0.05 level were considered significant.

## Results

**FIGURE 2:**
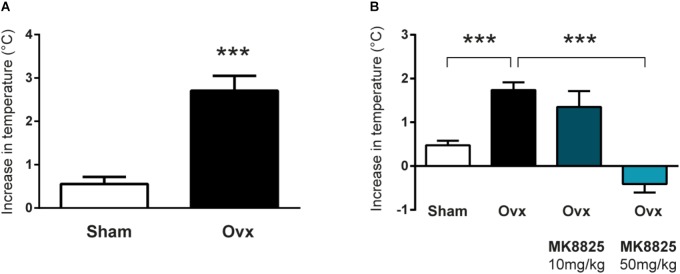
Flush-like increases in tail-skin temperature are inhibited by a CGRP receptor antagonist. (A) Increases in tail skin temperature induced by forced exercise in ovariectomized (Ovx; *n* = 6) and mice subjected to sham-surgery (Sham; *n* = 8). The change in temperature was calculated by subtracting the basal skin temperature from the temperature observed after forced exercise. (B) Changes in tail-skin temperature induced by forced exercise in mice treated with the CGRP receptor antagonist MK-8825 or saline (*n* = 13 + 13 +4 + 13). Results are displayed as mean ± SEM. Statistical significance is illustrated as ^∗∗∗^*P* < 0.001; Students *T*-test in **(A)** and 1-way ANOVA followed by Holm-Sidaks multiple comparisons test in **(B)**.

**FIGURE 3:**
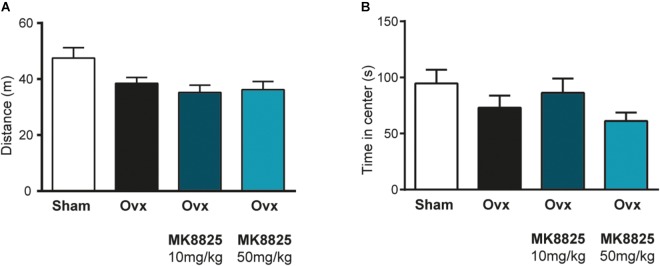
Normal locomotor activity and exploratory behavior after treatment with MK-8825. **(A)** Distance traveled during 15 min in an open field arena (*n* = 10 in all groups). Note that ovariectomized mice given MK-8825 show locomotor activity similar to oviarectomized mice given saline. **(B)** Time spent in the center of the open field arena. Results are displayed as mean ± SEM.

In order to study the role of CGRP in hot flushes, we used a previously described mouse model in which ovariectomized mice respond with abnormal increases in tail-skin temperature upon a short episode of exercise on a treadmill. A detailed time-line of the model is shown in Figure 1. We initially examined if we could reproduce the flush-like temperature increases reported with this model. Indeed, ovariectomized mice reacted with a much higher increase in tail skin temperature after exercise on the treadmill (2.70 ± 0.34°C, *n* = 6) compared to mice subjected to sham-surgery (0.56 ± 0.16°C, *n* = 8) (Figure 2A). Next, we investigated if administration of the non-peptide CGRP receptor antagonist MK-8825 affected the tail-skin temperature responses. Again, ovariectomized mice displayed an exaggerated tail-skin temperature increase (0.47 ± 0.11°C, *n* = 13) compared to control mice (1.74 ± 0.17°C, *n* = 13) (*p* < 0.0001) (Figure 2B). In contrast, ovariectomized mice that were treated with the CGRP receptor antagonist MK-8825 at a dose of 50 mg/kg exhibited no increase in tail skin temperature (-0.41 ± 0.19°C, *n* = 13) (*p* < 0.0001) (Figure 2B). Compared to sham operated animals, ovariectomized mice treated with MK-8825 at a dose of 50 mg/kg exhibited an altered temperature change (*p* = 0.0017). A lower dose of MK-8825 (10 mg/kg) did not significantly reduce the increase in tails skin temperature in ovariectomized mice (1.35 ± 0.37°C, *n* = 4) (*p* = 0.26). Compared to whereas the high dose of MK-8825 blocked the flush-like events, it should be noted that such an effect could reflect an inability to mount a response due to unspecific effects on the well-being of the mice. To examine if the mice were negatively affected in an unspecific way by the drug, we examined locomotor activity after the two doses of MK-8825. Mice treated with MK-8825 at a dose of 50 mg/kg did not display any obvious differences in locomotor activity (36.16 ± 2.99 m, *n* = 10) compared to Ovx mice given saline (38.45 ± 2.10 m, *n* = 10) (*p* = 0.90) (Figure 3A), which strongly indicates that the lack of flush-like events was unlikely to depend on a general effect on well-being. We also examined if MK-8825 affected exploratory behavior or induced anxiety-like behavior. This was done by quantifying the time the mice spent in the center of the open field during the open field test. As expected, MK-8825 did not affect the tendency to explore the central parts of the arena (Ovx + saline: 73.04 ± 10.72 s vs. Ovx + MK-8825: 61.23 ± 7.40 s; *p* = 0.79) (Figure 3B), which demonstrates that the drug is not affecting exploratory behavior or inducing anxiety-like behavior at a dose which efficiently blocked flush-like temperature changes (50 mg/kg).

**FIGURE 4:**
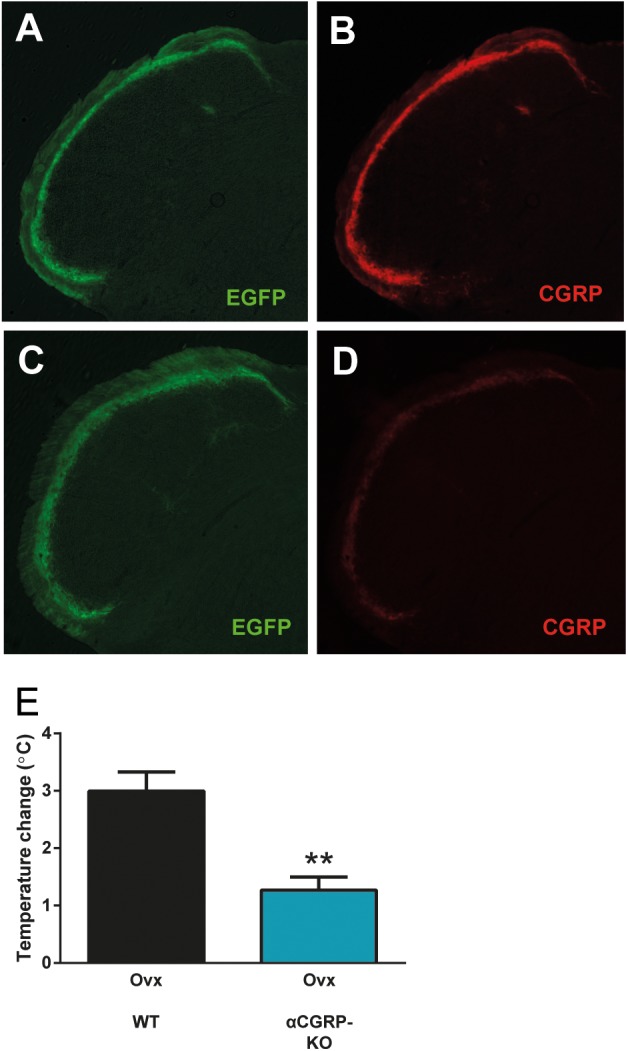
Attenuated flush-like events in mice lacking αCGRP. **(A,B)** Immunohistochemical detection of EGFP **(A)** and CGRP **(B)** in the spinal trigeminal nucleus in mice heterozygous for a construct in which the coding sequence of EGFP interrupts the gene encoding αCGRP and consequently abolishes αCGRP expression from the affected allele. Note that EGFP is expressed from the construct in a pattern identical to CGRP. **(C,D)** In mice homozygous for the construct (αCGRP-KO), strong EGFP labeling **(C)** but only very weak CGRP labeling **(D)** could be observed. **(E)** Temperature changes induced by forced physical activity in mice lacking αCGRP and normal mice from the same colony (*n* = 4 + 5). Results are displayed as mean ± SEM. Statistical significance is illustrated as ^∗∗^*P* < 0.01; Students *T*-test. Temperature changes induced by forced physical activity in mice lacking αCGRP and normal mice from the same colony (*n* = 4 + 5). Results are displayed as mean ± SEM. Statistical significance is illustrated as ^∗∗^*P* < 0.01; Students *T*-test.

To complement the pharmacological findings, we next used mice lacking αCGRP (McCoy et al., 2013), which is the form of CGRP that is predominant in the nervous system except for the enteric division. In these mice, the gene encoding αCGRP (and calcitonin) has been disrupted by a cassette expressing EGFP. Here, we did not use these features of the mouse-line, but simply used them as knock-outs for αCGRP. This genetic approach lacks the potential problems with non-specific activity that pharmacological agents have, and it allows a specific examination of the role of αCGRP, since the gene encoding βCGRP was left intact. As expected, we observed strong CGRP labeling in mice without the construct (data not shown) and in mice with the construct on only one allele (Figures 4A,B). The heterozygous mice displayed EGFP-expression from the construct in a pattern identical to that of CGRP. Mice homozygous for the construct displayed strong EGFP expression but very little CGRP immunoreactivity, which is not surprising since they should lack αCGRP (Figures 4C,D). As in the previous experiments, the control mice (ovariectomized mice from the same colony as the mice lacking αCGRP) displayed a marked increase in tail-skin temperature after exercise (3.00 ± 0.33°C, *n* = 4) (Figure 4E). This increase was markedly attenuated in ovariectomized mice lacking αCGRP (1.27 ± 0.23°C, *n* = 5) (*p* = 0.0031) (Figure 4E).

## Discussion

In this study, we found that administration of the CGRP receptor antagonist MK-8825 and deletion of αCGRP block or attenuate the exaggerated tail-skin temperature increases observed in ovariectomized mice after exercise. These findings complement earlier studies showing increased levels of CGRP in plasma during flushes in humans (Chen et al., 1993; Valentini et al., 1996; Wyon et al., 2000; Spetz et al., 2001).

Our findings clearly show that CGRP is important for hot flushes in a mouse model. However, the way by which CGRP is involved in the mechanisms underpinning hot flushes is still not clear. The fact that CGRP is a potent vasodilator and sweat gland activator, leads to flush-like symptoms when injected, and that it increases acutely in plasma during hot flushes may suggest that it is involved in the efferent link of the flush, i.e., that CGRP comes into play after the hypothalamus has initiated the flush-response (Padilla et al., 2018). Since CGRP is strongly expressed in primary afferents, in particular those of type C and Aδ, and these afferents often are closely associated with blood vessels, peripheral sensory nerves are a likely source of vasoactive CGRP. It is, however, not clear how the hypothalamus would trigger CGRP-release from sensory neurons. A recent study has identified kisspeptin and neurokinin B neurons in the arcuate nucleus of the hypothalamus as key players for the initiation of hot flushes, and neurokinin B release in the rostral preoptic area as a potential central mediator of the flush response in a mouse model for hot flushes (Padilla et al., 2018). Based on these findings, it may be hypothesized that neurokinin B release at the rostral preoptic hypothalamus initiates a signaling mechanism leading to peripheral release of CGRP. Another possibility is that CGRP is involved in the afferent component of the flush-response, i.e., that dysregulated CGRP-signaling is a part of the incoming information or the central computation that makes the hypothalamus trigger a flush. In this context, it is interesting to note that mice lacking αCGRP display increased body temperature (Walker et al., 2010). In addition, CGRP is expressed in central nervous pathways relaying temperature information. In particular, CGRP is strongly expressed in the external lateral part of the parabrachial nucleus (Paues et al., 2001), which is a critical hub in a pathway controlling body-temperature by providing information about skin cooling (Nakamura and Morrison, 2008). The fact that mice lacking αCGRP displayed reduced flush-like temperature increases indicates that αCGRP is involved. However, since the effect of the high dose of MK8825 was stronger than the effect of αCGRP deletion it is possible that βCGRP also plays a role. Another possibility is that this difference between the effect of the pharmacological and genetic interventions reflect the different genetic backgrounds of the mice used in the two experiments. Since the aim of the genetic experiment was to validate the finding that CGRP is important for flush-like temperature changes rather than dissecting the specific role of αCGRP, we used a minimalistic design and did not perform any further studies using more experimental groups or combinations of treatments.

Animal models of hot flushes have proved difficult to develop, but measurements of tail skin temperature in ovariectomized mice is an attractive model since it mirrors the peripheral vascular events seen in hot flushes (vasodilation in the skin leading to an increased temperature) and these effects can be blocked by estrogen supplementation (Kobayashi et al., 2000; Shuto et al., 2005). The model used in this study relies on flush-like events triggered by exercise on a treadmill. Whereas most hot flushes in humans come without obvious triggers, high impact exercise is one of the triggers known to induce flushes. This is not surprising since exercise generates heat and situations elevating core body temperature, such as exposure to warm ambient temperatures or intake of hot beverages, are common triggers for hot flushes. Regular exercise has been suggested to reduce the frequency of hot flushes by improving thermoregulatory control (Bailey et al., 2016), but current evidence does not support this claim (Daley et al., 2014; Lyon et al., 2018). With this in mind, it is important to note that the short episodes of physical activity that the mice were subjected to in this study were only used as flush-triggers and should not be sufficient to induce any general effects on thermoregulatory control. Even if there are obvious limitations caused by differences between human and animal physiology, we believe that this model has good potential for further studies of the mechanisms underlying the present findings. Since the final aim is to test the usefulness of interventions with CGRP signaling in humans with hot flushes, it will also be important to test antagonists, blocking antibodies or other forms of interventions in humans. Fortunately, drugs blocking CGRP signaling have been tested in humans, making clinical trials aimed at hot flushes both feasible and important.

## Author Contributions

DW, DE, ET, MH, and A-CS conceived the study. HD, HB, DW, AS, JZ, and EP performed the experiments. A-CS acquired the drug. DE supervised all animal experiments. DW, DE, and A-CS drafted the first version of the manuscript. All authors contributed substantially to the data analyses, revision of the manuscript and approved the final submitted version of the manuscript.

## Conflict of Interest Statement

The authors declare that the research was conducted in the absence of any commercial or financial relationships that could be construed as a potential conflict of interest.
